# Peripheral Blood Mitochondrial DNA as a Biomarker of Cerebral Mitochondrial Dysfunction following Traumatic Brain Injury in a Porcine Model

**DOI:** 10.1371/journal.pone.0130927

**Published:** 2015-06-22

**Authors:** Todd J. Kilbaugh, Maria Lvova, Michael Karlsson, Zhe Zhang, Jeremy Leipzig, Douglas C. Wallace, Susan S. Margulies

**Affiliations:** 1 Department of Anesthesiology and Critical Care Medicine, Children’s Hospital of Philadelphia, Perelman School of Medicine at the University of Pennsylvania, Philadelphia, Pennsylvania, United States of America; 2 Center for Mitochondrial and Epigenomic Medicine, Department of Pathology and Laboratory Medicine, Children’s Hospital of Philadelphia, Perelman School of Medicine at the University of Pennsylvania, Philadelphia, Pennsylvania, United States of America; 3 Mitochondrial Medicine, Department of Clinical Sciences, Lund University, Lund, Sweden; 4 Department of Bioengineering, University of Pennsylvania, Philadelphia, Pennsylvania, United States of America; St Michael's Hospital, University of Toronto, CANADA

## Abstract

**Background:**

Traumatic brain injury (TBI) has been shown to activate the peripheral innate immune system and systemic inflammatory response, possibly through the central release of damage associated molecular patterns (DAMPs). Our main purpose was to gain an initial understanding of the peripheral mitochondrial response following TBI, and how this response could be utilized to determine cerebral mitochondrial bioenergetics. We hypothesized that TBI would increase peripheral whole blood relative mtDNA copy number, and that these alterations would be associated with cerebral mitochondrial bioenergetics triggered by TBI.

**Methodology:**

Blood samples were obtained before, 6 h after, and 25 h after focal (controlled cortical impact injury: CCI) and diffuse (rapid non-impact rotational injury: RNR) TBI. PCR primers, unique to mtDNA, were identified by aligning segments of nuclear DNA (nDNA) to mtDNA, normalizing values to nuclear 16S rRNA, for a relative mtDNA copy number. Three unique mtDNA regions were selected, and PCR primers were designed within those regions, limited to 25-30 base pairs to further ensure sequence specificity, and measured utilizing qRT-PCR.

**Results:**

Mean relative mtDNA copy numbers increased significantly at 6 and 25 hrs after following both focal and diffuse traumatic brain injury. Specifically, the mean relative mtDNA copy number from three mitochondrial-specific regions pre-injury was 0.84 ± 0.05. At 6 and 25 h after diffuse non-impact TBI, mean mtDNA copy number was significantly higher: 2.07 ± 0.19 (P < 0.0001) and 2.37 ± 0.42 (P < 0.001), respectively. Following focal impact TBI, relative mtDNA copy number was also significantly higher, 1.35 ± 0.12 (P < 0.0001) at 25 hours. Alterations in mitochondrial respiration in the hippocampus and cortex post-TBI correlated with changes in the relative mtDNA copy number measured in peripheral blood.

**Conclusions:**

Alterations in peripheral blood relative mtDNA copy numbers may be a novel biosignature of cerebral mitochondrial bioenergetics with exciting translational potential for non-invasive diagnostic and interventional studies.

## Introduction

Estimates predict that traumatic brain injury (TBI) will become the third leading cause of death and disability in the world by 2020 [1]. TBI is a heterogeneous insult to the brain induced by traumatic biomechanical forces. TBI precipitates a complex, secondary pathophysiological process which can result in a cascade of deleterious side effects often far from the site of the initial injury, which places tissue that survives the initial insult at risk for functional failure, neurodegeneration, apoptosis, and death [2–5]. Critical secondary pathophysiologic pathways following TBI are modulated by mitochondrial dysfunction and activation of the innate immune system in the brain as well as peripherally [6]. Our knowledge of the role of the innate immune system in response to TBI in immature animals is limited [7].

Microglia, present throughout the CNS, comprise the main component of the innate immune system within the brain, which, under pathologic conditions are activated and increase production of ROS (via nicotinamide adenine dinucleotide phosphate (NADPH) oxidase (NOX), a multi-subunit enzyme activated in host defense), nitrogen species (RNS), and expression inducible isoform of nitric oxide synthase (iNOS), and pro-inflammatory cytokines that propagate secondary brain injury [8,9] [10]. One way microglia may be activated is by damage associated molecular patterns (DAMPs) that are recognized by pattern recognition receptors (PRR) on the surface of microglia themselves and astrocytes. Microglial activation and neuroinflammatory production of ROS may cause additional mitochondrial oxidative phosphorylation dysfunction, furthering ROS generation and proinflammatory cytokine expression, all of which may feedback to cause further damage to essential components of mitochondria and release mitochondrial damage-associated proteins (MTD), such as formyl peptides and mitochondrial DNA (mtDNA), into the extracellular milieu in the brain [11]. MTD have been shown to activate the peripheral innate immune system via white blood cells (WBC) activation through formyl peptide receptor 1 and toll like receptor 9 (TLR-9), triggering a sterile systemic inflammatory response by activating WBC [12]. However, to our knowledge, little is known about the activation of the peripheral innate immune system secondary to acute brain injury, and whether this injury triggers alterations in mitochondrial biogenesis and free circulating extracellular mtDNA copy numbers in the immature animal. Furthermore, while there are no peripheral biomarkers in routine clinical use, mtDNA content holds promise as a global biomarker for the prediction of global alterations in mitochondrial dysfunction from acquired injury [13] [14].

Therefore, we hypothesized that isolated traumatic brain injury would increase peripheral whole blood mtDNA concentrations following TBI. In addition, we hypothesized that the increase in mtDNA content measured in the peripheral blood would correlate with alterations in cerebral mitochondrial bioenergetic function at varying time points following TBI, as a potential peripheral biosignature of TBI, expanding the current group of biomarkers available for study, combination, and clinical use.

## Materials and Methods

This study was carried out in strict accordance with the Guide for the Care and Use of Laboratory Animals of the National Institutes of Health. The protocol was approved by the Institutional Animal Care and Use Committee of the University of Pennsylvania. All surgeries were performed under general anesthesia and all efforts were made to minimize suffering. Four-week-old piglets (8–10 kg), which have comparable neurodevelopment to a human toddler, were used for the study [15,16]. Females were chosen to limit heterogeneity between genders based on prior work [17]. Thirteen piglets were designated into two injury cohorts: 1) controlled cortical impact at the rostal gyrus (n = 5 injured-CCI), 2) single rapid non-impact rotational injury in the sagittal plane (n = 8 injured-RNR). Injured animals were sacrificed at 6 (n = 4) and 25 (n = 4) hours after RNR and at 25 hours after CCI. Pre-injury and post-injury blood samples were acquired by intravenous phlebotomy prior to injuries and whole blood was separated into 200 μL aliquots and stored at -80 degrees Celsius. A group of sixteen sham animals (n = 6 naïve sham-CCI and n = 10 naïve sham-RNR) were used to acquire baseline ex-vivo mitochondrial respiration in brain regions specific to injury type. These values were then used to determine the change effect in cerebral mitochondrial respiration in individual injured animals. The difference in sham and cerebral mitochondrial respiration in individual animals was then correlated to each individual animal’s change in relativel mtDNA copy number measured in peripheral blood from pre- and post-injury.

### Animal preparation

Piglets were premedicated with an intramuscular injection of ketamine (20 mg/kg) and xylazine (2 mg/kg) followed by 4% inhaled isoflurane in 1.0 fraction of inspired oxygen via snout mask, until abolishment of response to a reflexive pinch stimulus. Endotracheal intubation was followed by a decrease in fraction of inspired oxygen to 0.21 and maintenance of anesthesia with 1% inhaled isoflurane. Buprenorphine (0.02 mg/kg) was delivered intramuscularly for analgesia prior to injury. Using a heating pad, core body temperature was kept constant between 36 and 38°C and monitored via a rectal probe. Non-invasive blood pressure, oxygen saturation, heart rate, respiratory rate, and end-tidal CO_2_ were continuously monitored throughout the experiment (VetCap model 2050081; SDI, Waukesha, WI). If necessary, mechanical ventilation was utilized to maintain normoxia and normocarbia before injury, otherwise piglets maintained spontaneous ventilation.

### Controlled Cortical Impact (CCI) Injury

The right coronal suture was exposed, while maintained on isoflurane anesthetic, and a craniectomy was performed over the rostral gyrus allowing a 1 cm margin around the indentor tip of the cortical impact device described previously [18]. The exposed dura was opened in a stellate fashion to reveal the cortical surface, and the device was stabilized against the skull with screws. The spring-loaded tip rapidly (4ms) indented 0.63 cm of the cortical rostral gyrus [18]. The device was removed, the dura re-approximated, and the surgical flap sutured closed.

### Rapid Non-impact Rotational (RNR) Injury

Diffuse closed head TBI was induced using an established rapid head rotation technique described previously [19]. While maintained on isoflurane, the head of the piglet was secured to a bite plate by a snout strap. Isoflurane was withdrawn immediately prior to injury, and the head was rotated rapidly (10–15 ms) ventral-to-dorsal in the sagittal plane with the center of rotation at the cervical spine.

Animals were extubated following injuries. Both CCI and RNR animals were recovered when they met the following criteria: vocalization without squealing, able to ambulate despite some persistent gait instability, absence of piloerection, and proper feeding and drinking behaviors. Animals were hypoactive and spent more time recumbent position than non-injured littermates following injury, but were able to drink and eat unassisted. Animals that were sacrificed at 6 hours remained in the laboratory and those sacrificed at 25 hours returned to the animal housing facility. These injuries are best described as mild-to-moderate in severity, based on parallels with human clinical severity classifications [20,21].

### Mitochondrial High-Resolution Respirometry (HRR)

In the CCI injured animals a measured 2 cm^2^ region of cortex and underlying white matter was resected immediately adjacent to the rostral edge of the contusion, along with a corresponding 2 cm^2^ region from the contralateral hemisphere. In the RNR injured animals, a 2 cm^2^ region of left frontal cortex was resected and both hippocampal regions were extracted and combined. For both CCI and RNR samples, tissue was removed within seconds, and placed in ice-cold isolation buffer (320 mM sucrose, 10 mM Trizma base, and 2 mM EGTA), where blood, blood vessels, or necrotic tissue were dissected and disposed. In addition, subcortical white matter was removed from the cortex. A final concentration of tissue homogenate with MiR05 analyzed was 1 mg/ml at a constant 37°C, and the rate of oxygen consumption, was measured utilizing a high-resolution oxygraph and was expressed in pmols/[s*mg of tissue homogenate], (OROBOROS, Oxygraph-2k and DatLab software all from OROBOROS Instruments, Innsbruck, Austria). The oxygraph was calibrated daily, and oxygen concentration was automatically calculated from barometric pressure and MiR05 oxygen solubility factor set at 0.92 relative to pure water. A substrate-uncoupler-inhibitor titration (SUIT) protocol previously used for rodent brain tissue was further developed and optimized for studies in porcine brain tissue [22]. Sequential additions were used to acquire a detailed representation of mitochondrial respiration. Respiratory capacities with electron flow through both complex I (CI) and complex II (CII) were evaluated separately as well as the convergent electron input through the Q-junction (CI + II) using succinate and nicotinamide adenine dinucleotide (NADH)-linked substrates [23]. Succinate (10 mM) was added to stimulate maximal oxidative phosphorylating respiration capacity via convergent input through complexes I and II (OXPHOS_CI+CII_). Oligomycin, an inhibitor of ATP-synthase, induced mitochondrial respiration independent of ATP production across the inner mitochondrial membrane, commonly referred to as LEAK respiration (LEAK_CI+CII_) or State 4_O_.

All animals were sacrificed for fresh brain tissue, required for mitochondrial analysis, precluding neuro-pathologic evaluation; however, in a group of animals with CCI and RNR injuries of a similar magnitude and survival duration, we observe consistent, characteristic focal and diffuse patterns of injury [24], and functional alterations [25].

### Bioinformatics

A bioinformatics procedure was designed to identify PCR primers unique to mtDNA. The swine reference genome was downloaded from Ensembl (Sscrofa10.2). Segments of the mtDNA were aligned to nDNA to identify regions including sequences unique to mtDNA. Three such regions were selected within three mitochondrial coding genes: mitochondrial cytochrome c oxidase subunit (COI-1) and I-A (COI-1A); as well as, Nicotinamide adenine dinucleotide dehydrogenase (NADH) subunit 4 (ND4), a core subunit of complex I in the mitochondrial membrane respiratory system. Primer3 (http://biotools.umassmed.edu/bioapps/primer3_ww.cgi) was then used to pick pairs of PCR primers within those regions, devoid of nuclear mitochondrial DNA (NUMT). The lengths of all primers were limited to 25–30 base pairs to further ensure sequence specificity.

### Relative Quantitative real-time PCR (RQ-PCR)

Total genomic DNA was extracted from whole blood aliquots using Qiagen DNeasy Blood and Tissue Kit (Cat. No. 69506) according to the manufacturer’s protocol. Quality of DNA was assayed using Qubit Fluorometer (Invitrogen). To quantify mtDNA copy number in peripheral blood, qRT-PCR was performed using TaqMan reagents. 3 Custom Taqman Gene Expression Assays (primer-probe-primer) were designed, one laying in ND4 gene and 2 in COI gene. ND4 primers: forward 5’-ATAGCCTATCCATTCCTCATGCTTT, reverse 5’-GTGTACTCGTTCATAGTTAGTGTTGG; probe: AGGCATAATCATAACCAGCTC. COI primers: forward 5’-AACTGACTCGTACCGCTAATAATCG, reverse 5’-TGAGGATGCCAGAAGTAATAGGAAG; probe 1 CTTTCCACGTATAAACAAC; probe 2 CTTTCCACGTATAAACAACA. DNA was amplified in a final volume of 20 μl, using 1.0 μl of 20x Taqman Assay, 10.0 μl of Taqman Gene Expression Master Mix (Life Technologies) and 20 ng of total DNA. The amplification was performed under universal conditions on Viia 7 RT-PCR Platform (Life Technologies). Viia 7 software V.1.2.3 was used for the analysis. All samples were analyzed in triplicate. To quantify target sequences, comparative C_T_ (ΔΔC_T_) was used relative to a reference sample and displayed as a relative quantification ratio. The relative quantification mitochondrial copy number (RQ) was defined, for each gene, as the ratio of mtDNA gene copy number to an internal control of nuclear DNA (nDNA), 16S rRNA qRT-PCR; resulting in mtDNA/nDNA ratio. The relative quantification mitochondrial copy number (RQ) is proportional to mtDNA copy number in each sample.

### Statistical analysis

Comparisons for the change effect of pre-injury and post-injury mtDNA RQ measurements were evaluated with a Wilcoxon matched-pair sign rank test for each gene and combined analysis using PRISM 6.0 software (GraphPad Software Inc., La Jolla, CA, USA). Intergroup delta relative mtDNA copy number changes for each gene were compared using repeated-measures analysis of variance (RM-ANOVA) followed by multiple comparison procedures. Differences between post-injury groups (6 hours and 25 hours post-RNR) were compared with Mann-Whitney *U* Test.

Oxygen flux traces were analyzed with a customized Matlab (Mathworks, Natick, MA) program designed to extract the oxygen flux at each phase of the SUIT protocol. The correlation between the differences between a subject’s pre- and post-TBI relative mtDNA copy number, and the difference between average sham cerebral mitochondrial respiratory values and the subject’s post-TBI cerebral mitochondrial respiratory values was assessed separately for CCI and RNR animals using Pearson’s correlation coefficient. A value of P ≤ 0.05 was considered statistically significant. In the results, the means and the standard errors of means are presented.

## Results

### Alterations in peripheral blood mtDNA copy number 6 and 25 hours after diffuse and 25 hours after focal traumatic brain injury

**Fig 1:**
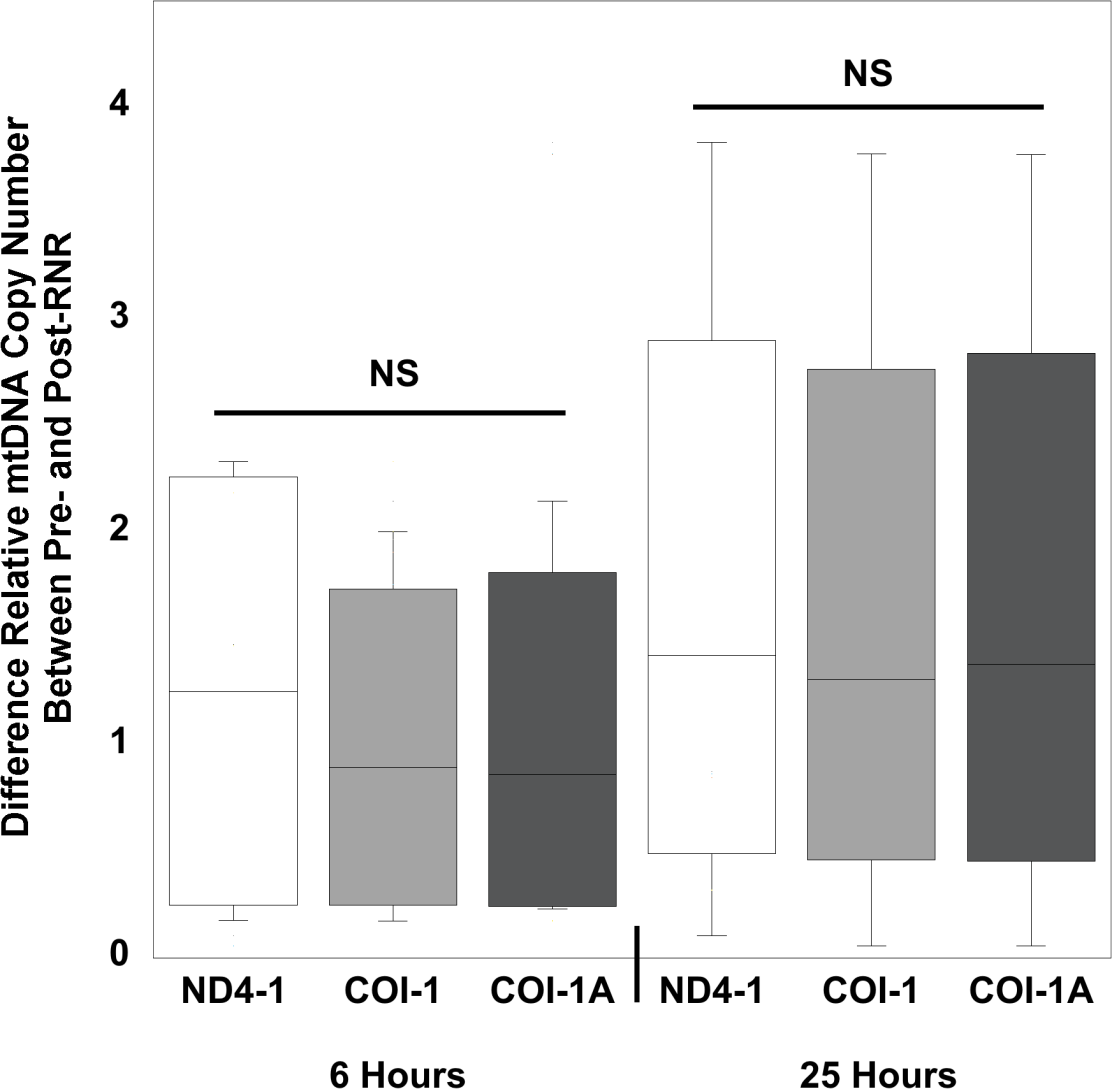
Relative quantification values of mtDNA concentrations for each gene measured from pre-injury to 6 hours and 25 hours post-injury in diffuse, rapid non-impact rotational (RNR) traumatic brain injury. Box plot analysis illustrating the difference in relative quantification values of mtDNA concentrations measured in peripheral whole blood for each gene measured from pre-injury to 6 and 25 hours post-RNR. Increases in all three genes measured were similar following RNR at both time points. Nicotinamide adenine dinuclueotide dehydrogenase (NADH) subunit 4mitochondrial (ND4-1). Cytochrome c oxidase subunit (COI-1) and I-A (COI-1A). All values are mean ± SEM.

**Fig 2:**
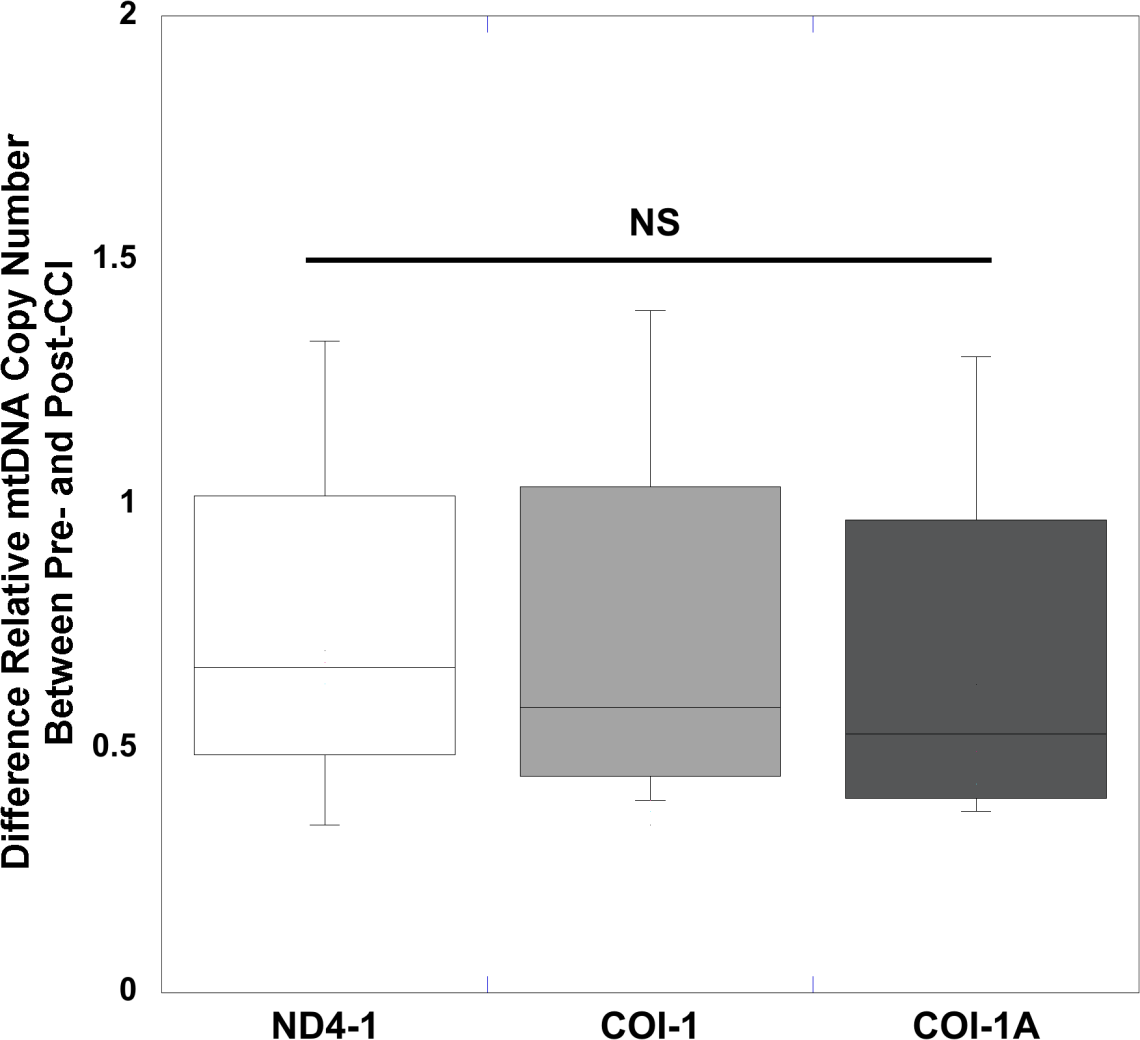
Relative quantification values of mtDNA concentrations measured in peripheral whole blood for each gene measured from pre-injury to post-injury in focal, controlled cortical impact (CCI) traumatic brain injury. Box plot analysis illustrating the difference in relative quantification values of mtDNA concentrations measured in peripheral whole blood for each gene measured from pre-injury to 25 hours post-CCI. Increases in all three genes measured were similar following CCI at 25 hours. Nicotinamide adenine dinuclueotide dehydrogenase (NADH) subunit 4mitochondrial (ND4-1). Cytochrome c oxidase subunit (COI-1) and I-A (COI-1A). All values are mean ± SEM.

**Fig 3:**
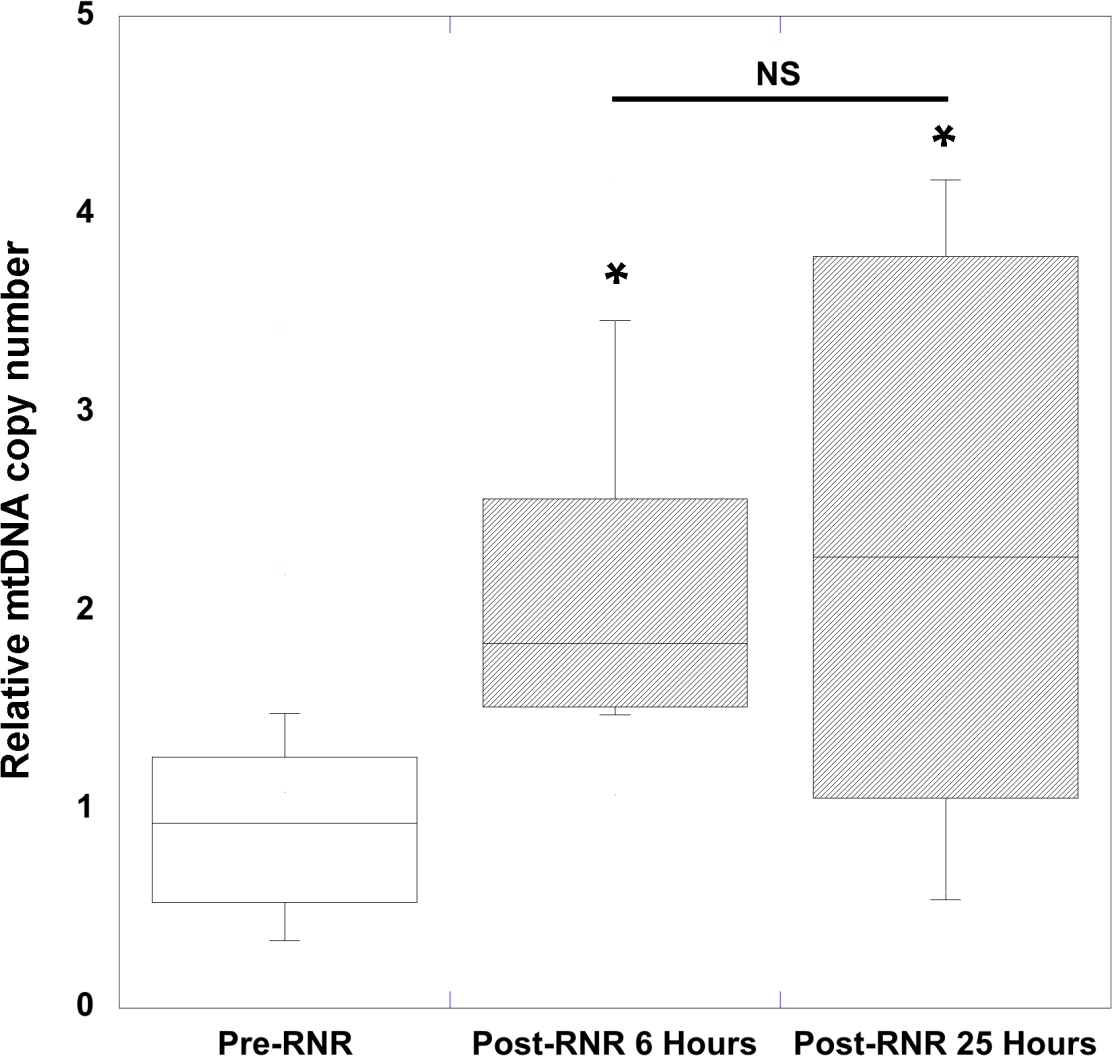
Peripheral blood mtDNA copy number in rapid non-impact rotational (RNR) traumatic brain injury. RNR traumatic brain injury resulted in significant increases in relative quantification mitochondrial copy number (RQ) measured in peripheral whole blood at 6 hours and 25 hours post-RNR. All values are the mean of three specific mitochondrial genes measured (ND4-1, COI-1, and COI-1A) and pooled together ± SEM. Nicotinamide adenine dinuclueotide dehydrogenase (NADH) subunit 4mitochondrial (ND4-1). Cytochrome c oxidase subunit (COI-1) and I-A (COI-1A). *P<0.001 vs. Pre-RNR.

**Fig 4:**
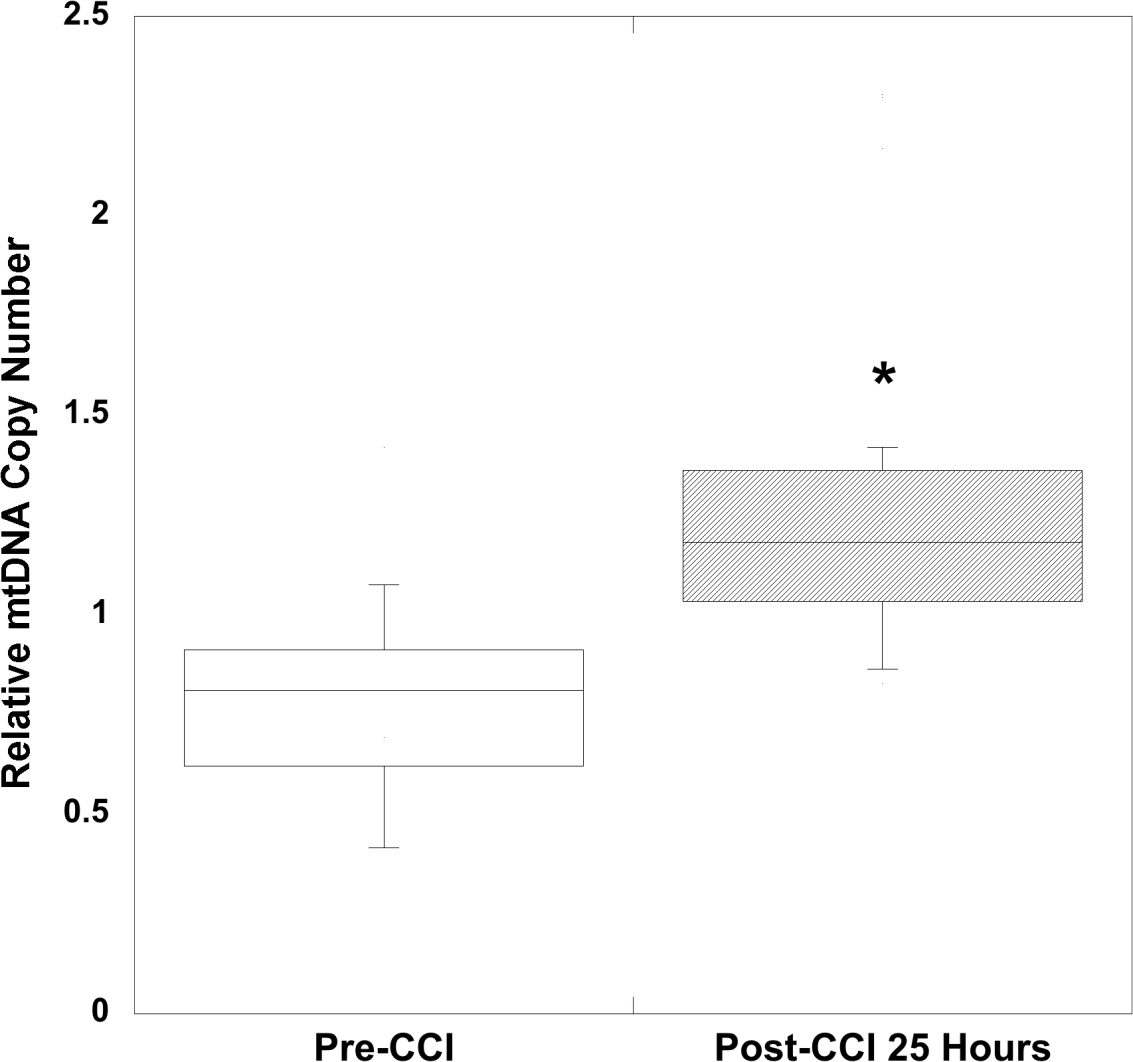
Peripheral blood mtDNA copy number in focal, controlled cortical impact (CCI) traumatic brain injury. CCI traumatic brain injury resulted in significant increases in relative quantification mitochondrial copy number (RQ) measured in peripheral whole blood at 25 hours post-RNR. All values are the mean of three specific mitochondrial genes measured (ND4-1, COI-1, and COI-1A) pooled together ± SEM. Nicotinamide adenine dinuclueotide dehydrogenase (NADH) subunit 4mitochondrial (ND4-1). Cytochrome c oxidase subunit (COI-1) and I-A (COI-1A). *P<0.0001 vs. Pre-CCI.

Mean peripheral blood mtDNA copy number was significantly increased after focal (25 hours) and diffuse (6 hours and 25 hours) traumatic brain injury compared to pre-injury levels. Using relative quantitative PCR techniques to measure relative mtDNA copy number, for 2 genes devoid of NUMT determined by bioinformatics analysis, we measured the relative content determined by the ratio of mtDNA/16S rRNA with respect to the COI and ND4-1 genes in peripheral blood, following TBI from diffuse (Table 1) and focal injuries (Table 2). To determine the pre-injury average relative mtDNA copy number across all animals, we first determined that there was no statistical difference between pre-injury relative mtDNA copy number across all genes measured in RNR animals (n = 24) and CCI animals (n = 15) (P = 0.29, Mann Whitney *U* test). In addition, there was no difference in the delta change between pre- and post- relative mtDNA copy number across the 3 genes at either 6 (P = 0.86) or 25 hours (P = 0.95), or between the two time points (P = 0.94, repeated-measures ANOVA) for diffuse RNR injuries (Fig 1). Similarly, there were no differences in the delta changes across all three genes 25 hours after focal CCI (P = 0.98) (Fig 2). Therefore, we defined the pre-injury relative mtDNA copy number by pooling together the RQ of all genes obtained from all animals to determine the average pre-injury mean, 0.84 ± 0.05 (n = 39). Next, we pooled the post-injury relative mtDNA copy number across all genes for each injury group and time point, and then compared these values to the pooled pre-injury relative mtDNA copy number. We found that there was a significant increase in relative mtDNA copy number 6 hours after diffuse RNR TBI (n = 12), 2.07 ± 0.19 (P < 0.0001, Wilcoxon signed rank test) (Fig 3). Furthermore, 25 hours post-RNR the relative mtDNA copy number (n = 12) 2.37 ± 0.42 (P = 0.0005) was significantly increased compared to pre-injury levels. There was no significant difference in relative mtDNA copy number between time points measured at 6 and 25 hours post-RNR (P = 0.62, Mann-Whitney *U* test) (Fig 3). Similarly, the relative mtDNA copy number (n = 15) 25 hours post-injury was significantly increased, 1.35 ± 0.12 (P < 0.0001) compared to pre-injury relative mtDNA copy number (Fig 4).

**Table 1:** Levels of mtDNA content in peripheral blood WBC pre- and post-RNR TBI at 6 hours and 25 hours.

mtDNA	Pre-Injury	6 Hours Post-RNR	Pre-Injury	25 Hours Post-RNR
COI-1	0.99±0.16	1.99±0.34	0.68±0.19	2.30±0.79
COI-1A	0.99±0.16	1.99±0.34	072±0.21	2.34±0.80
ND4-1	1.24±0.1	2.22±0.44	0.71±0.23	2.44±0.83

Mean ± SEM C_T_ quantifications from real-time PCR assays of *COI-1*, *COI-1A*, *ND4-1* amplicons from peripheral whole blood. Relative mtDNA copy number was determined, for each gene, by the ratio of mtDNA gene copy number to an internal control of nuclear DNA (nDNA), to a 16S rRNA qRT-PCR. The RQ ratio is proportional to mtDNA copy number in each cell. Nicotinamide adenine dinuclueotide dehydrogenase (NADH) subunit 4mitochondrial (ND4-1). Cytochrome c oxidase subunit (COI-1) and I-A (COI-1A).

**Table 2:** Levels of mtDNA content in peripheral blood WBC pre- and post-CCI TBI at 25 hours.

mtDNA	Pre-Injury	25 Hours Post-CCI
COI-1	0.75±0.11	1.33±0.25
COI-1A	0.75±0.09	1.29±0.23
ND4-1	0.79±0.11	1.43±0.23

Mean ± SEM C_T_ quantifications from real-time PCR assays of *COI-1*, *COI-1A*, *ND4-1* amplicons from peripheral whole blood. Relative mtDNA copy number was determined, for each gene, by the ratio of mtDNA gene copy number to an internal control of nuclear DNA (nDNA), to a 16S rRNA qRT-PCR. The RQ ratio is proportional to mtDNA copy number in each cell. Nicotinamide adenine dinuclueotide dehydrogenase (NADH) subunit 4mitochondrial (ND4-1). Cytochrome c oxidase subunit (COI-1) and I-A (COI-1A).

### Relationship of mtDNA copy number and cerebral mitochondrial bioenergetics following traumatic brain injury

To investigate whether increases in relative mtDNA copy number measured in peripheral blood correlated with alterations in cerebral mitochondrial bioenergetics, we compared cerebral mitochondrial respiration findings from tissue homogenates following traumatic brain injury. We then correlated the differences between pre- and post-TBI relative mtDNA copy number measured in peripheral blood in both injuries to differences between sham and post-TBI cerebral mitochondrial respiration to determine the predictive value of relative mtDNA copy number on determining disordered cerebral mitochondrial bioenergetics following TBI.

Previously, we studied alterations in mitochondrial respiration following TBI injury in both injury models and found that there are significant reductions in hippocampal maximal oxidative phosphorylation (OXPHOS_CI+CII_) measured by HRR at 6 hours and a significant decrease in the respiratory control ratio (RCR) OXPHOS_CI+CII_ (State 3)/LEAK_CI+CII_ (State 4_O_) at 25 hours in diffuse RNR compared to sham animals. In our model of CCI, peri-contusional tissue in the ipsilateral side of the focal injury displayed a significant reduction in maximal oxidative phosphorylation (OXPHOS_CI+CII_), with significant uncoupling of respiration. Thus, these previous experiments (data not shown) informed our selection of these respiratory states at particular time points to correlate with the change in peripheral relative mtDNA copy number. Additionally, sham animals were used to determine baseline respiration of each respiratory state because experiments are conducted on fresh tissue, rendering it impossible to measure pre- and post-TBI tissue respiration. The average pre-injury hippocampal OXPHOS_CI+CII_ respiration and RCR used for differences between pre- and post-injury calculations with RNR were, respectively: 82.6 ± 5.9 pmols O_2_/s*mg and 5.91. The average sham cortical OXPHOS_CI+CII_ used for differences between pre- and post-injury calculations with CCI was 76.0 ± 4.0 pmols O_2_/s*mg.

**Fig 5:**
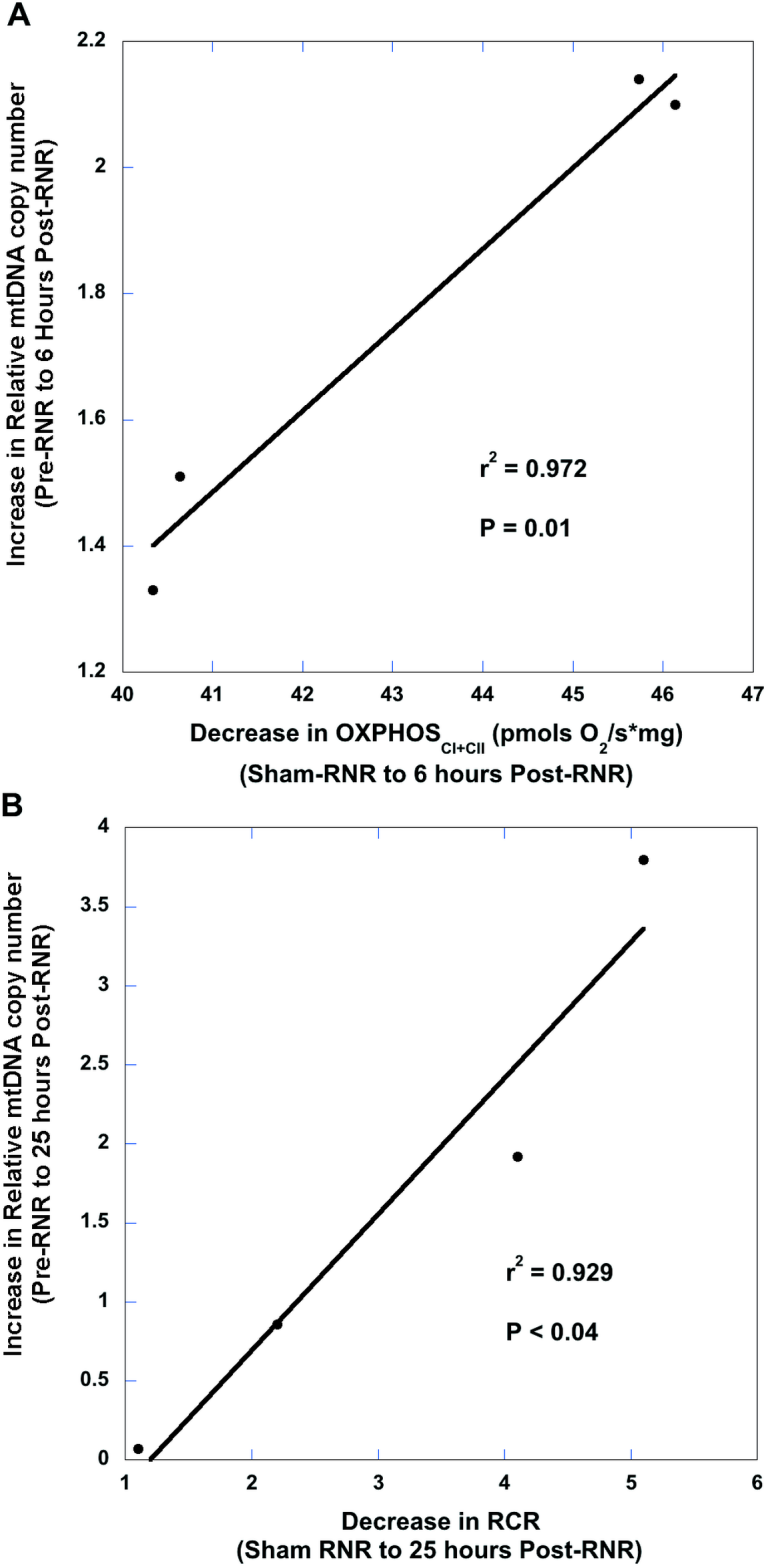
Association between peripheral blood mtDNA copy number and cerebral bioenergetics 6 hours and 25 hours after rapid, non-impact rotational (RNR) traumatic brain injury. (A) There was a significant correlation between the increase in peripheral blood relative mtDNA quantification copy number (RQ), from pre- to post-injury and the decrease in hippocampal maximal oxidative phosphorylation 6 hours post-RNR from sham respiration values. RNR: rapid non-impact rotational traumatic brain injury. (B) There was a significant correlation between the increase in peripheral blood relative mtDNA quantification copy number (RQ), from pre- to post-injury and the decrease in hippocampal maximal oxidative phosphorylation (OXPHOS_CI+CII_) 25 hours post-RNR from sham respiration. RNR: rapid non-impact rotational traumatic brain injury.

**Fig 6:**
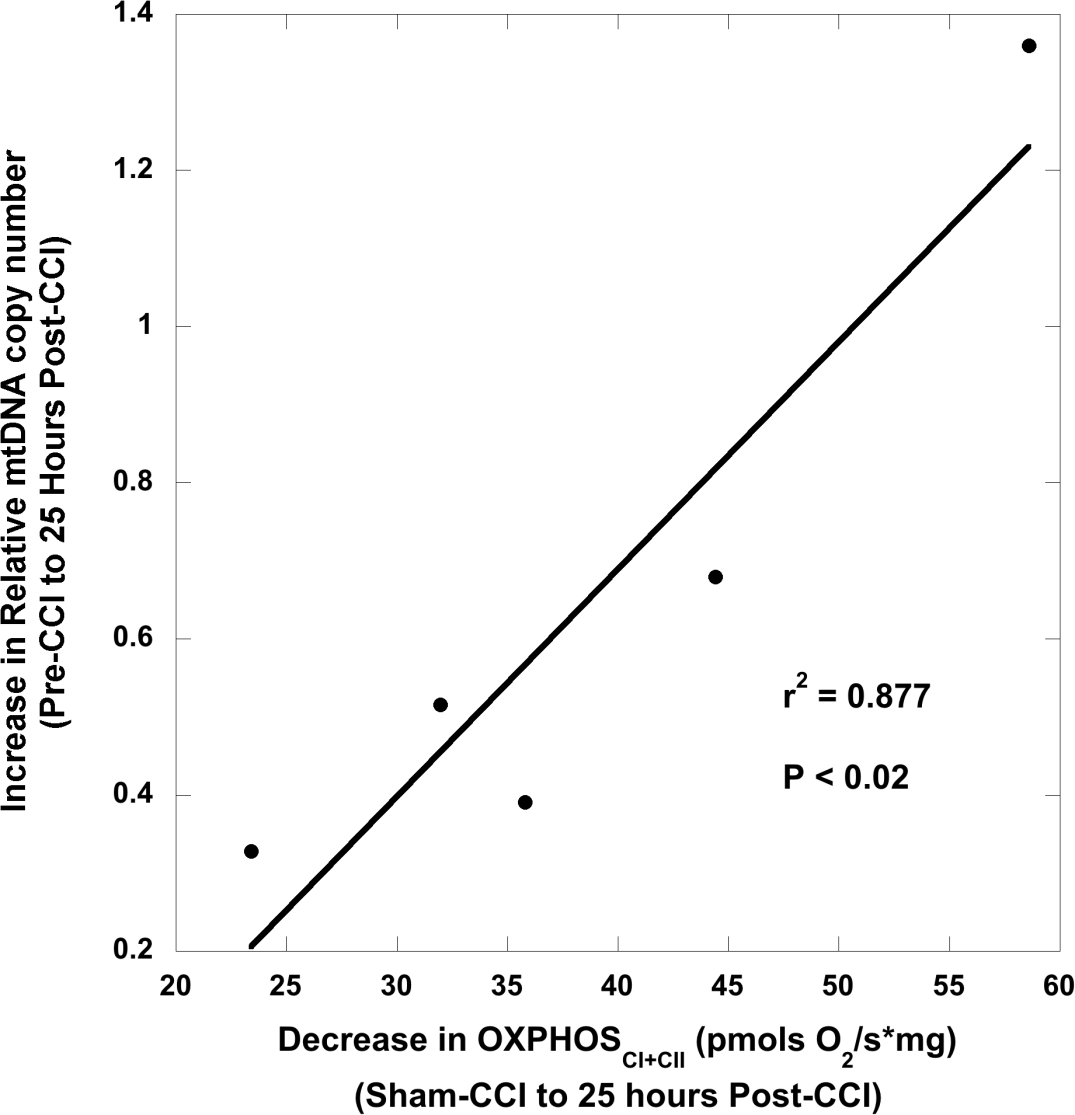
Association between peripheral blood mtDNA copy number and cerebral bioenergetics 25 hours after controlled cortical impact (CCI) traumatic brain injury. There was a significant correlation between the increase in peripheral blood relative mtDNA quantification copy number (RQ), from pre- to post-injury and the decrease in maximal oxidative phosphorylation (OXPHOS_CI+CII_) in peri-contusional CCI tissue 25 hours post-CCI from sham respiration. CCI: controlled cortical impact traumatic brain injury.

Six hours after TBI we found that larger increases in relative mtDNA copy number in peripheral blood after RNR from pre-injury concentrations were significantly correlated with lower maximal oxidative phosphorylation (OXPHOS_CI+CII_; pmols O_2_/s*mg) in the hippocampus (P <0.01) (Fig 5A) compared to sham respiration. Furthermore, there was a significant correlation 25 hours post-RNR between increases in relative mtDNA copy number and lower hippocampal RCR values (P < 0.04) (Fig 5B). Animals who underwent CCI were found to have a significant correlation 25 hours after injury between increased relative mtDNA copy number and lower maximal oxidative phosphorylation (OXPHOS_CI+CII_; pmols O_2_/s*mg) in the peri-contusional tissue in the ipsilateral hemisphere (P < 0.02) (Fig 6) compared to sham respiration.

## Discussion

In two high-fidelity models of immature traumatic brain injury we established that relative mitochondrial DNA copy number from peripheral whole blood samples significantly increased at 6 and 25 hours following isolated brain injury compared to pre-injury concentrations. Importantly, to the best of our knowledge, this is the first study to observe a statistical correlation between increased peripheral blood relative mtDNA copy number and alterations in brain mitochondrial bioenergetics following traumatic brain injury.

### Response of the peripheral immune system to TBI

Pathogens trigger the innate immune response through a cascade initiated by pattern recognition receptors (PRR) [26]. These receptors recognize conserved structures from microbes, known as pathogen associated molecular patterns (PAMPs), and in turn trigger gene transcription and stimulate the innate immune response, including a systemic inflammatory response syndrome (SIRS) [26]. It is becoming increasingly recognized that cellular injury from trauma can elicit a similar sterile SIRS when the same PRRs that respond to PAMPs, sense endogenous molecular structures, known as damage (or danger) associated molecular patterns (DAMPs) [27]. Mitochondrial DAMPs (MTD), formyl peptides and mtDNA, are two well conversed molecular signatures that retain molecular motifs similar to their bacterial ancestors that act on PRR, such as formyl peptide receptor 1 and toll-like receptor 9 (TLR-9), and can initiate an inflammatory response modulated by leukocyte activation [28]. Mitochondrial DNA is extremely susceptible to oxidative damage for several reasons, including its proximity to high concentrations of reactive oxygen species (ROS) generated within the electron transport system, and that it is devoid of protective histone proteins [29]. Significant increases in peripheral MTD, has been observed in trauma patients following major injuries. In addition experimental rodent models intravenously-injected with MTD displayed an activated innate immunity precipitating marked inflammatory organ injury [12]. Furthermore, Walko and colleagues found that children with severe traumatic brain injury (defined by those with Glasgow Coma Scales (GCS) scores less than 8 who required intracranial pressure monitoring via an external ventricular drain) had significantly higher mtDNA copy numbers in cerebral spinal fluid following injury compared to controls [30].

We have shown that in large animal models of mild-to-moderate isolated TBI isolated head injury can increase the relative mtDNA copy number in peripheral blood following injury to the immature brain. We speculate that following TBI and the disruption of the blood brain barrier (BBB), DAMPs are released that cross the BBB into the peripheral circulation that trigger PRRs, increasing the relative mtDNA copy number in the peripheral blood, which is likely a combination of leukocyte mitochondrial biogenesis and an increase in free mtDNA released following central injury [31,32]. This likely represents that activation of the innate immune system and propagation of a systemic inflammatory response following traumatic brain injury [33]. In addition, activation of the peripheral innate immune system, as seen in this study, may then feed back and propagate inflammation in the injured brain with peripheral inflammatory cells and cytokines crossing the damaged BBB and triggering microglial activation [34].

### Serum biosignature of traumatic brain injury

Serologic markers of TBI for outcome prediction and interpretation of complex molecular cascades following primary TBI is critical for prognostication, time course delineation, and interpretation of therapeutic interventions [14]. Unfortunately, there has been a lack of discovery and development of novel biomarkers for pediatric TBI, and none that provide a potential link to ongoing cerebral mitochondrial dysfunction [13]. In addition, the routine use of computed tomography (CT) and magnetic resonance imaging (MRI) is problematic in terms of cost, radiation exposure, and logistics, and may not reveal structural abnormalities even in symptomatic patients; therefore, easily obtained peripheral biosignatures of ongoing alterations in cerebral bioenergetics may provide critical information in patients in a patient population thought to be severely under diagnosed [35]. In this study of mild-to-moderate TBI we found a significant correlation between increases in relative mtDNA copy number in peripheral whole blood and alterations in cerebral bioenergetics following diffuse and focal injury. Specifically, we found that increases in relative mtDNA copy number correlated with reductions in hippocampal maximal oxidative phosphorylation at 6 hours and increased mitochondrial uncoupling at 25 hours after diffuse RNR injury, as well as reductions in maximal oxidative phosphorylation in peri-contusional tissue following focal CCI at 25 hours. Alterations in mitochondrial bioenergetics may play a significant role in propagation of secondary injury cascades following TBI due to cellular inability to maintain energy output, limit reactive oxygen species (ROS), buffer calcium, inhibit apoptosis, and limit the release of MTD [4]. Previously we have shown that mitochondrial dysfunction at 6 (data not shown) and 24 hours is associated with significant injury measured by neuropathology in both models [36]. It is difficult to study alterations in mitochondrial bioenergetics, including alterations in oxidative phosphorylation and uncoupling of the inner mitochondrial membrane, because sampling requires ex-vivo tissue analysis or global surrogate of measures mitochondrial metabolism from invasive neuromonitoring such as lactate pyruvate ratios from cerebral microdialysis (MD). Thus, the use of relative mtDNA copy number in peripheral whole blood samples at different time signatures may be a novel, minimally, invasive peripheral biosignature of pediatric TBI and cerebral bioenergetic dysfunction. Peripheral whole blood mtDNA copy numbers hold significant translational promise because pre-injury levels displayed little variability measured by ANOVA across all animals, qRT-PCR techniques are readily available, and peripheral whole blood is easily obtained and rapidly processed, even in patients with mild and moderate injury who do not require invasive neuromonitoring or intensive care management.

### Limitations

Our understanding of mechanisms responsible for the association between traumatic injury in the immature brain and increased peripheral relative mtDNA copy number in peripheral whole blood is limited. Future studies should correlate values in peripheral blood with those in the CSF, and identify the causal mechanisms triggered by MTD released from the injured brain into the peripheral circulation that activate this specific innate immune response. In addition, the relationship between whole blood mtDNA, free mtDNA, and clonal leukocyte expansion should be explored further.

## Conclusion

In conclusion, we find rapid increases of relative mtDNA copy numbers in peripheral blood following isolated focal and diffuse TBI. Because larger increases in relative mtDNA copy numbers correlated significantly with lower levels of cerebral tissue mitochondrial respiration following injury, relative mtDNA copy numbers in peripheral blood may provide a unique opportunity for translation as a novel biosignature of cerebral bioenergetic dysfunction following TBI. Future studies should investigate mechanisms responsible, including peripheral immune system responses, of mitochondrial danger signaling initiated by central nervous system trauma.
